# Combining the dynamic model and deep neural networks to identify the intensity of interventions during COVID-19 pandemic

**DOI:** 10.1371/journal.pcbi.1011535

**Published:** 2023-10-18

**Authors:** Mengqi He, Sanyi Tang, Yanni Xiao

**Affiliations:** 1 School of Mathematics and Statistics, Shaanxi Normal University, Xi’an, China; 2 School of Mathematics and Statistics, Xi’an Jiaotong University, Xi’an, China; University of Washington, UNITED STATES

## Abstract

During the COVID-19 pandemic, control measures, especially massive contact tracing following prompt quarantine and isolation, play an important role in mitigating the disease spread, and quantifying the dynamic contact rate and quarantine rate and estimate their impacts remain challenging. To precisely quantify the intensity of interventions, we develop the mechanism of physics-informed neural network (PINN) to propose the extended transmission-dynamics-informed neural network (TDINN) algorithm by combining scattered observational data with deep learning and epidemic models. The TDINN algorithm can not only avoid assuming the specific rate functions in advance but also make neural networks follow the rules of epidemic systems in the process of learning. We show that the proposed algorithm can fit the multi-source epidemic data in Xi’an, Guangzhou and Yangzhou cities well, and moreover reconstruct the epidemic development trend in Hainan and Xinjiang with incomplete reported data. We inferred the temporal evolution patterns of contact/quarantine rates, selected the best combination from the family of functions to accurately simulate the contact/quarantine time series learned by TDINN algorithm, and consequently reconstructed the epidemic process. The selected rate functions based on the time series inferred by deep learning have epidemiologically reasonable meanings. In addition, the proposed TDINN algorithm has also been verified by COVID-19 epidemic data with multiple waves in Liaoning province and shows good performance. We find the significant fluctuations in estimated contact/quarantine rates, and a feedback loop between the strengthening/relaxation of intervention strategies and the recurrence of the outbreaks. Moreover, the findings show that there is diversity in the shape of the temporal evolution curves of the inferred contact/quarantine rates in the considered regions, which indicates variation in the intensity of control strategies adopted in various regions.

## Author summary

When applying the compartment model to simulate the disease transmission dynamics, some parameters or particular functions are assumed to describe the intensity of the control interventions. However, these preset specific functions may not accurately quantify the intervention strategies, which brings great challenges to accurately make prediction and evaluation. In this study, we developed an extended transmission-dynamics-informed neural network algorithm by integrating deep neural network with epidemic model. Even for insufficient case data, the proposed algorithm can still help us reconstruct the temporal evolution trend of the epidemic and infer unknown parameters. We inferred the time series on contract rate and quarantine rate for six regions based on the case data, on which the reasonable and interpretable functions, describing the dynamic variation in the intensity of control strategies, can be successfully selected and determined. The inferred contact/quarantine rates in various regions show the diverse shapes and regional dependent, and hence the variation in the intensity of control measures. This suggests the dynamic zero-case policy exhibits the different efficacy in reducing contacts and increasing the quarantine and isolation.

## Introduction

The COVID-19 pandemic has lasted for three years since the end of 2019. Due to the continuous variation of the virus strain and the dynamic adjustment of prevention and control measures, it is a great challenge to propose a dynamic model of infectious diseases to evaluate the effectiveness of non-pharmaceutical interventions (NPIs) [1]. In particular, before October 2022, due to China’s implementation of the dynamic zero-case policy, strong close contact tracing and isolation measures or even static management mode make almost all outbreaks be cleared in about 40 days. From the point view of mathematical modelling, increased quarantine/isolation rate and decreased contact rate have played an essential role in reducing new infections. However accurately quantifying the rate functions and examining their effects on infections remain unclear and fall within the scope of this study.

Modelling the dynamics of infectious diseases is an essential tool to provide the quantitative basis for decision making during the COVID-19 pandemic. Traditionally, the intrinsic transmission mechanism of infectious diseases and the flow among individuals in various compartments are mainly described by ordinary/partial differential equations [2, 3], delay differential equations [4] and fractional differential equations [5]. In traditional mechanism-based models, researchers usually incorporated constant contact rate and quarantine/isolation rate for simplicity to analyze the transmission risk [6], model the impact of contact tracing and quarantine on the development of COVID-19 [7, 8] and evaluate the independent effectiveness of vaccines [9]. There are also a large number of literatures in which the specific functions were supposed to represent the dynamic changes in intensity of interventions for comparing the effectiveness of various control strategies [10], understanding the drivers of multiple waves of outbreaks [11] and exploring the transmission mechanism of COVID-19 with different intervention patterns [12]. Moreover, Wang et al. [13] considered a dynamic epidemiological model with a piecewise contact rate and quarantine rate to simulate the dynamics of the Omicron variant in Shanghai, and explored the feasibility of different control patterns in avoiding subsequent waves. Li et al. [14] developed a model with pulse population-wide nucleic acid screening, and simulated the changes of contact/quarantine rates over time by using exponential decline/increase functions, respectively, focusing on the impact of large-scale screening on the transmission dynamics of COVID-19 infection and the operation of medical resources. Note that the preset specific functions may not accurately capture the dynamic adjustment of intervention strategies. And the assumed rate functions may inevitably involve more parameters in the model, which brings significant challenges to data fitting and parameter estimation. This mechanism of preset rate functions inevitably causes that the outcomes are usually dependent on the particular types of rate functions due to various assumptions. Hence the data-driven inference of rate function is of great significance to quantify and assess the intensity of control interventions.

Data-driven statistical models are widely used in biological, medicine, social science and other fields due to the flexibility and feasibility of the method [15, 16]. Especially in recent years, it has played an important role in simulating COVID-19 pandemic [17, 18]. For example, Sindhu et al. [19] proposed a three parametric model named as Exponentiated transformation of Gumbel Type-II (ETGT-II) for analyzing the number of deaths due to COVID-19 for Europe and China. In addition, there are several studies have developed different types of statistical models based on COVID-19 mortality data and evaluated the performance of the models [20, 21]. Rahman et al. [22] developed a seasonal Autoregressive Integrated Moving Average (ARIMA) model and eXtreme Gradient Boosting (XGBoost) model to simulate the overall trend of confirmed cases and deaths of COVID-19 infection in Bangladesh, and compared the accuracy of predictions of two methods. Külah et al. [23] considered Shifted Gaussian Mixture Model with Similarity-based Estimation (SGSE) to predict the development trend of COVID-19 pandemic for a specific country by examining similar behaviors in other countries. Note that these data-driven statistical methods do not incorporate prior transmission mechanisms, resulting in poor interpretability of simulation results, making it difficult to provide decision-making basis for optimizing control strategies.

Data-driven deep learning is another powerful tool for analyzing the dynamics of COVID-19 pandemic. It is a nonlinear mathematical tool with powerful learning ability, and is widely used in natural language processing [24], fault detections [25], image recognitions [26] and reliability analysis [27–29]. During COVID-19 pandemic, neural networks are used to construct various simulation frameworks to predict the development trend of the epidemic [30, 31]. For example, Jin et al. [32] predicted COVID-19 infection based on multiple neural networks and reinforcement learning. Shafiq et al. [33] estimated the COVID-19 mortality rates in Italy by using maximum likelihood estimation and artificial neural network (ANN). Xu et al. [34] employed three different deep learning models, including the convolutional neural network (CNN), long short-term memory (LSTM) and convolutional neural network-long short-term memory (CNN-LSTM), to predict the number of new cases and forecast the spread of COVID-19 infection. Utku [35] developed a convolutional neural network-gated recurrent unit (CNN-GRU), based on hybrid deep learning model, to predict COVID-19 cross-country spread. Gautam [36] applied transfer learning to the LSTM network to learn the trends of new cases and new death of COVID-19 infection from case data in Italy and the United States and to make projections for other countries. However, the black box attribute of the algorithms makes it face uninterpretable risks, especially the end-to-end learning method cannot reveal the underlying transmission mechanism of epidemics or the impact of intervention measures on mitigating the disease spread.

The main purpose of this study is to combine scattered observational data with deep learning and epidemic models, in order to avoid assuming the specific rate functions in advance and make neural networks follow the rules of epidemic systems in the process of learning. This mechanism of physics-informed neural network (PINN) may provide a flexible computational framework for scientific problems [37, 38]. By applying a data-driven module to extend an epidemiological model with control interventions derived from first principles, we implement the time-dependent parameters that quantify the intensity of prevention and control measures as different neural networks, and then embed the epidemiological model into the neural network through adding the residuals of the equations to the loss function, and develop an extended transmission-dynamics-informed neural network algorithm framework. We simulate the COVID-19 epidemic evolution trend in Xi’an, Guangzhou, Yangzhou, Hainan and Xinjiang with TDINN, and discover the temporal evolution pattern of time-dependent parameters reflecting the dynamic adjustment of the control strategies based on the epidemic curves in these regions. Furthermore, we reconstruct the dynamic evolution trend of time-dependent parameters through specific functions and provide interpretability analysis for the output of deep learning. Finally, We also test the fitting performance of the TDINN algorithm on the COVID-19 infection with multiple waves in Liaoning province.

In this study, we will develop a TDINN algorithm that integrates epidemic data, deep learning, and epidemiological models to identify the intensity of interventions during COVID-19 pandemic. It is worth noting that the TDINN algorithm guides neural networks to adhere to epidemic system rules during the learning process and meanwhile avoids the pre-assumption of modeling contact/quarantine rates with specific functions. The proposed algorithm can not only fit the multi-source epidemic data well, but also reconstruct the epidemic development trend with incomplete reported data. Further, we successfully trace the temporal evolution patterns of the contact rate and quarantine rate, and perform the interpretability analysis of the time-dependent rate functions inferred by TDINN algorithm.

## Methods

### Data

**Fig 1:**
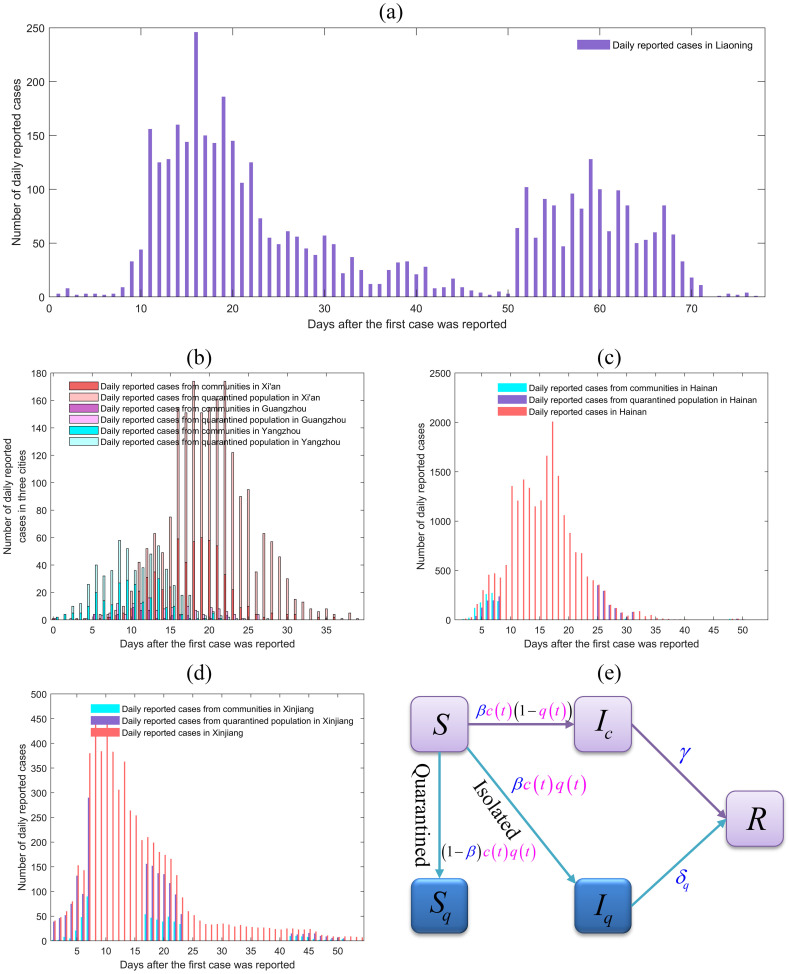
Multi-source epidemic data and the framework of transmission dynamic model. (a) Epidemic data of COVID-19 infection in Liaoning province from 6th March 2022 to 21st May 2022; (b) Epidemic data of COVID-19 infection in Xi’an from 9th December 2021 to 20th January 2022, in Guangzhou from 21st May to 18th June 2021, and in Yangzhou from 28th July to 26th August 2021; (c) Epidemic data of COVID-19 infection in Hainan from August 1st to September 23rd, 2022; (d) Epidemic data of COVID-19 infection in Xinjiang from August 4th to September 26th, 2022; (e) Flow diagram among epidemiological classes.

We obtained the daily reported number of confirmed cases for Xi’an outbreak from December 9th, 2021 to January 20th, 2022, Guangzhou outbreak from May 21st, 2021 to June 18th, 2021, and for Yangzhou outbreak from July 28th, 2021 to August 26th, 2021 from Health Commissions of Shaanxi [39], Guangdong [40] and Jiangsu provinces [41], respectively. In addition, we collected the daily reported number of confirmed cases from August 1st, 2022 to September 23rd, 2022 in Hainan province [42] and form August 4th, 2022 to September 26th, 2022 in Xinjiang Uygur Autonomous Region [43], respectively. Data information includes the number of daily reported cases in the community(${I_{c}}_{\text{new}}^{data}\left(t\right)$) and in the quarantined zone(${I_{q}}_{\text{new}}^{data}\left(t\right)$). It is important to note that the numbers of daily reported case in the community or quarantined zone are incomplete for Hainan and Xinjiang, but we have complete daily reported case numbers (${I_{r}}_{\text{new}}^{data}\left(t\right)$) in these two regions. Moreover, we also obtained the daily reported number of confirmed cases for Liaoning outbreak from 6th March 2022 to 21st May 2022 from Health Commissions of Liaoning provinces [44], where the data information only contains a column of daily reported case numbers (${I_{r}}_{\text{new}}^{data}\left(t\right)$) and shows multi-wave outbreaks. Detailed data are shown in Fig 1a–1d.

For Xi’an, Guangzhou, and Yangzhou, we can calculate the cumulative reported cases in the community(${I_{c}}_{\text{cum}}^{data}\left(t\right)$)(or quarantined zone (${I_{q}}_{\text{cum}}^{data}\left(t\right)$)) based on the daily reported cases in the community (or quarantined zone), while for Hainan, Xinjiang and Liaoning, we only obtain the cumulative reported cases(${I_{r}}_{\text{cum}}^{data}\left(t\right)$). Therefore, in this study, we have access to three categories of reported data sets, which are as the follows:

- Set 1: ${I_{c}}_{\text{new}}^{data}\left(t\right)$, ${I_{q}}_{\text{new}}^{data}\left(t\right)$, ${I_{c}}_{\text{cum}}^{data}\left(t\right)$, ${I_{q}}_{\text{cum}}^{data}\left(t\right)$, for Xi’an, Guangzhou, Yangzhou;
- Set 2: ${I_{c}}_{\text{new}}^{data}\left(t\right)$, ${I_{q}}_{\text{new}}^{data}\left(t\right)$, ${I_{r}}_{\text{new}}^{data}\left(t\right)$, ${I_{r}}_{\text{cum}}^{data}\left(t\right)$, for Hainan, Xinjiang;
- Set 3: ${I_{r}}_{\text{new}}^{data}\left(t\right)$, ${I_{r}}_{\text{cum}}^{data}\left(t\right)$, for Liaoning.

### The model

During COVID-19 pandemic, China’s government has mainly adopted the dynamic zero-case policy, i.e., strict close contact tracking and isolation, high-frequency and large-scale nucleic acid screening, closed management and etc, to quickly respond to the outbreak. These powerful NPIs effectively make most infected people not go through the complete process from infection to incubation period, and then to asymptomatic or symptomatic, that is, patients may be detected at every stage after infection. Therefore, this study simulates the transmission mode and evolution dynamics of COVID-19 infection based on the classic deterministic Susceptible-Infected-Removed (SIR) type epidemiological model [2]. Then we extend the simplest SIR-type dynamic model by including contact tracing and isolation, and the flow diagram is shown in Fig 1e. Given an outbreak taking off in a city, the city can usually be divided into two regions according to different intensity of control measures: free region (or community) and quarantined region. Consequently, we stratify the population in the free (quarantined) region into the susceptible class *S* (*S*_*q*_) and the infected class *I*_*c*_ (*I*_*q*_), and the removed class (denoted by *R*). Note that here we do not distinguish the individuals in the removed class in free or quarantined region since they can not be re-infected within a relatively short duration, and then consider a single compartment. Here we use the subscripts ‘*q*’ to represent quarantined population, i.e. *S*_*q*_ and *I*_*q*_ represent quarantined susceptible class and quarantined infected class, respectively. To model the continuously adjusted intervention measures, we assume the time-dependent contact rate and quarantine rate, denoted by *c*(*t*) and *q*(*t*), respectively. The transmission probability of per contact is supposed to be *β*. Then, the quarantined individuals, if infected (or uninfected), move to the compartment *I*_*q*_ (or *S*_*q*_) at a rate of *βc*(*t*)*q*(*t*) (or (1 − *β*)*c*(*t*)*q*(*t*)). Those who are not quarantined, if infected, will move to the compartment *I*_*c*_ at a rate of *βc*(*t*)(1 − *q*(*t*)). According to the fact that the quarantined individuals do not return to the susceptible population before the end of outbreak, then we ignore the rate of transition from *S*_*q*_ to *S* class. Hence we have the following ordinary differential equations:

$$\begin{array}{c} \left\{ \begin{array}{cc} \frac{\text{d}S}{\text{d}t} & =-\frac{\beta c(t)+c(t)q(t)(1-\beta )}{N}SI_{c},\\ \frac{\text{d}I_{c}}{\text{d}t} & =\frac{\beta c(t)(1-q(t))}{N}SI_{c}-\gamma I_{c},\\ \frac{\text{d}S_{q}}{\text{d}t} & =\frac{\left(1-\beta )c(t)q(t\right)}{N}SI_{c},\\ \frac{\text{d}I_{q}}{\text{d}t} & =\frac{\beta c(t)q(t)}{N}SI_{c}-\delta_{q}I_{q},\\ \frac{\text{d}R}{\text{d}t} & =\gamma I_{c}+\delta_{q}I_{q}, \end{array}\right. \end{array}$$ (1)

where *N* represents the total population of the region, the recovery rate of infected individuals in community (quarantined region) denoted by *γ*(*δ*_*q*_), and the definitions and values of all parameters used in the model are given in Table 1. Here we consider three additional auxiliary compartments to record cumulative reported cases ($I_{r_{\text{cum}}}$), the cumulative reported cases in the community ($I_{c_{\text{cum}}}$) (or the quarantined region ($I_{q_{\text{cum}}}$)). The dynamics of these three compartments are driven by the following equations:

$$\begin{array}{c} \left\{ \begin{array}{cc} \frac{\text{d}I_{c_{\text{cum}}}}{\text{d}t} & =\frac{\beta c(t)(1-q(t))}{N}SI_{c},\\ \frac{\text{d}I_{q_{\text{cum}}}}{\text{d}t} & =\frac{\beta c(t)q(t)}{N}SI_{c},\\ \frac{\text{d}I_{r_{\text{cum}}}}{\text{d}t} & =\frac{\beta c(t)}{N}SI_{c}. \end{array}\right. \end{array}$$ (2)

**Table 1:** Parameter definitions and estimation for model (1).

Parameter	Definitions	Estimated values						Sources
		Xi’an	Guangzhou	Yangzhou	Hainan	Xinjiang	Liaoning	
*c*(*t*)	Time-dependent contact rate	See text and Fig 3c	See text and Fig 3g	See text and Fig 3k	See text and Fig 4d	See text and Fig 4i	See text and Fig 7b	TDINN
*q*(*t*)	Time-dependent quarantine rate	See text and Fig 3d	See text and Fig 3h	See text and Fig 3m	See text and Fig 4e	See text and Fig 4j	See text and Fig 7c	TDINN
*β*	Probability of transmission per contact	0.1498	0.1893	0.1493	0.1281	0.1977	0.2544	TDINN
*γ*	Recovery rate of community infected individuals	0.2953	0.2337	0.2994	0.2830	0.1773	0.3691	TDINN
*δ*_*q*_	Recovery rate of quarantined infected individuals	0.3531	0.2507	0.1950	0.2737	0.3519	0.2155	TDINN

### Parameter estimation

It is known that fully connected deep neural networks with arbitrary nonlinear activation functions are universal approximators [45], we then use three independent neural networks with time *t* as input to represent the time-dependent contact rate *c*(*t*), quarantined rate *q*(*t*) and each state variable in model (1) respectively. So we have

$$\begin{array}{c} c(t)=c^{\text{NN}}(t;\Theta_{c}),\;\;q(t)=q^{\text{NN}}(t;\Theta_{q}),\;\;U(t)=U^{\text{NN}}(t;\Theta_{U}), \end{array}$$

where *U* is a vector of all epidemiological categories considered in model (1), i.e., *U* = (*S*, *I*_*c*_, *S*_*q*_, *I*_*q*_, *R*). Here *c*^NN^, *q*^NN^, *U*^NN^ represent neural network operators and (Θ_*c*_, Θ_*q*_, Θ_*U*_) is a parameter set composed of network weights and biases.

**Fig 2:**
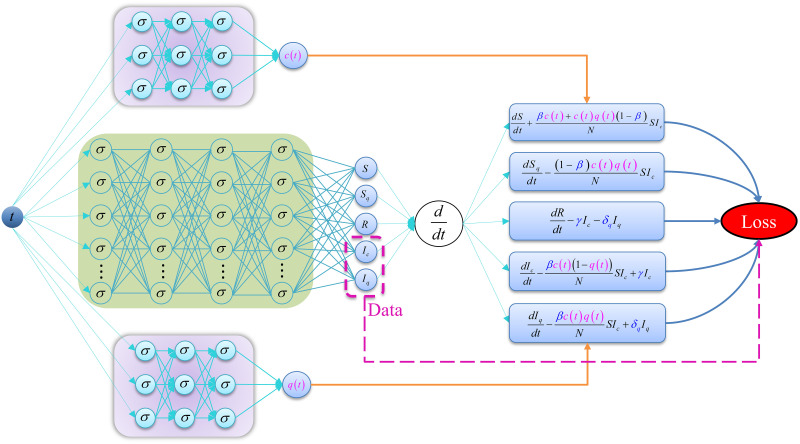
Schematic diagram of transmission-dynamics-informed neural network. Different neural networks are used to represent the state variables (green shaded area) and time-dependent parameters (purple shaded area) of model (1). The symbols “*σ*” and “$\frac{d}{dt}$” represent the activation function and the automatic differentiation operator, respectively.

Based on the method of physics-informed neural networks proposed in [37], we integrate three different neural networks to obtain an extended transmission-dynamics-informed neural network(TDINN), shown in Fig 2. The neural networks in the purple shaded area are used to infer the time-dependent contact rate *c*(*t*) and quarantine rate *q*(*t*). The neural network in the green shaded area is used to fit the available data and approximately solve model (1). The approximated network solution of model (1) can be defined as

$$U^{\text{NN}}(t)=(S^{\text{NN}}(t),{I_{c}}^{\text{NN}}(t),{S_{q}}^{\text{NN}}(t),{I_{q}}^{\text{NN}}(t),R^{\text{NN}}(t)).$$

The next critical step is to embed the information of transmission dynamics into the neural network to constrain the output(solutions) to satisfy the observational data and the ODE system, which is achieved by constructing a loss function corresponding to reported data and epidemiological models. Specifically, the output of the neural network at the temporal nodes $\{t_{d}^{i}{\}}_{i=1}^{T_{d}}$ should be as close as possible to the observed data. In addition, we enforce the neural network to satisfy the ODE system at the temporal nodes $\{t_{e}^{i}{\}}_{i=1}^{T_{e}}$. This can be achieved by using automatic differentiation to calculate the residual error of the ODE system at $\{t_{e}^{i}{\}}_{i=1}^{T_{e}}$, so $\{t_{e}^{i}{\}}_{i=1}^{T_{e}}$ is also called “residual points”. Here, let $T_{d}$ represent the number of observed data and $T_{e}$ represent the number of residual points. It is worth noting that residual points can be arbitrarily sampled in the entire computational domain. To measure the mismatch between the outputs from neural network/ODE systems and the observed data, we define the loss function as follows [37]:

$$\begin{array}{c} Loss={\text{MSE}}_{data}+{\text{MSE}}_{ode}, \end{array}$$ (3)

where MSE stands for mean square error, MSE_*data*_ is used to measure the degree of matching between the output of the neural network and the observed data, and MSE_*ode*_, as a penalty term, describes whether the solution learned by the neural network satisfies the ODE system.

The first term MSE_*data*_ in the loss function (3) has different expressions based on the three categories of available datasets. For the data in Set 1,

$$\begin{array}{c} \begin{array}{cc} {\text{MSE}}_{data} & =\frac{1}{T_{d}}\sum_{i=1}^{T_{d}}{\left|{I_{c}}_{\text{new}}^{\text{NN}}(t_{d}^{i})-{I_{c}}_{\text{new}}^{data}(t_{d}^{i})\right|}^{2}+\frac{1}{T_{d}}\sum_{i=1}^{T_{d}}{\left|{I_{q}}_{\text{new}}^{\text{NN}}(t_{d}^{i})-{I_{q}}_{\text{new}}^{data}(t_{d}^{i})\right|}^{2}\\ & +\frac{1}{T_{d}}\sum_{i=1}^{T_{d}}{\left|{I_{c}}_{\text{cum}}^{\text{NN}}(t_{d}^{i})-{I_{c}}_{\text{cum}}^{data}(t_{d}^{i})\right|}^{2}+\frac{1}{T_{d}}\sum_{i=1}^{T_{d}}{\left|{I_{q}}_{\text{cum}}^{\text{NN}}(t_{d}^{i})-{I_{q}}_{\text{cum}}^{data}(t_{d}^{i})\right|}^{2}. \end{array} \end{array}$$

For the data in Set 2, the MSE_*data*_ becomes

$$\begin{array}{c} \begin{array}{cc} {\text{MSE}}_{data} & =\frac{1}{T_{d}}\sum_{i=1}^{T_{d}}{\left|{I_{c}}_{\text{new}}^{\text{NN}}(t_{d}^{i})-{I_{c}}_{\text{new}}^{data}(t_{d}^{i})\right|}^{2}+\frac{1}{T_{d}}\sum_{i=1}^{T_{d}}{\left|{I_{q}}_{\text{new}}^{\text{NN}}(t_{d}^{i})-{I_{q}}_{\text{new}}^{data}(t_{d}^{i})\right|}^{2}\\ & +\frac{1}{T_{d}}\sum_{i=1}^{T_{d}}{\left|{I_{r}}_{\text{new}}^{\text{NN}}(t_{d}^{i})-{I_{r}}_{\text{new}}^{data}(t_{d}^{i})\right|}^{2}+\frac{1}{T_{d}}\sum_{i=1}^{T_{d}}{\left|{I_{r}}_{\text{cum}}^{\text{NN}}(t_{d}^{i})-{I_{r}}_{\text{cum}}^{data}(t_{d}^{i})\right|}^{2}. \end{array} \end{array}$$

For the data in Set 3, the MSE_*data*_ becomes

$$\begin{array}{c} \begin{array}{c} {\text{MSE}}_{data}=\frac{1}{T_{d}}\sum_{i=1}^{T_{d}}{\left|{I_{r}}_{\text{new}}^{\text{NN}}(t_{d}^{i})-{I_{r}}_{\text{new}}^{data}(t_{d}^{i})\right|}^{2}+\frac{1}{T_{d}}\sum_{i=1}^{T_{d}}{\left|{I_{r}}_{\text{cum}}^{\text{NN}}(t_{d}^{i})-{I_{r}}_{\text{cum}}^{data}(t_{d}^{i})\right|}^{2}. \end{array} \end{array}$$

Where ${I_{c}}_{\text{new}}^{\text{NN}}$, ${I_{q}}_{\text{new}}^{\text{NN}}$, ${I_{r}}_{\text{new}}^{\text{NN}}$, ${I_{c}}_{\text{cum}}^{\text{NN}}$, ${I_{q}}_{\text{cum}}^{\text{NN}}$ and ${I_{r}}_{\text{cum}}^{\text{NN}}$ represent the approximate solution of the neural network.

Combining ordinary differential Eqs (1) and (2), we give the residual form of each component as follows:

$$\begin{array}{c} \begin{array}{cc} L_{1}\left(t_{e}^{i}\right) & =\frac{\text{d}}{\text{dt}}S^{\text{NN}}\left(t_{e}^{i}\right)+\frac{\beta c^{\text{NN}}\left(t_{e}^{i}\right)+c^{\text{NN}}\left(t_{e}^{i}\right)q^{\text{NN}}\left(t_{e}^{i}\right)\left(1-\beta \right)}{N}S^{\text{NN}}\left(t_{e}^{i}\right){I_{c}}^{\text{NN}}\left(t_{e}^{i}\right),\\ L_{2}\left(t_{e}^{i}\right) & =\frac{\text{d}}{\text{dt}}{I_{c}}^{\text{NN}}\left(t_{e}^{i}\right)-\frac{\beta c^{\text{NN}}\left(t_{e}^{i}\right)\left(1-q^{\text{NN}}\left(t_{e}^{i}\right)\right)}{N}S^{\text{NN}}\left(t_{e}^{i}\right){I_{c}}^{\text{NN}}\left(t_{e}^{i}\right)+\gamma {I_{c}}^{\text{NN}}\left(t_{e}^{i}\right),\\ L_{3}\left(t_{e}^{i}\right) & =\frac{\text{d}}{\text{dt}}{S_{q}}^{\text{NN}}\left(t_{e}^{i}\right)-\frac{\left(1-\beta \right)c^{\text{NN}}\left(t_{e}^{i}\right)q^{\text{NN}}\left(t_{e}^{i}\right)}{N}S^{\text{NN}}\left(t_{e}^{i}\right){I_{c}}^{\text{NN}}\left(t_{e}^{i}\right),\\ L_{4}\left(t_{e}^{i}\right) & =\frac{\text{d}}{\text{dt}}{I_{q}}^{\text{NN}}\left(t_{e}^{i}\right)-\frac{\beta c^{\text{NN}}\left(t_{e}^{i}\right)q^{\text{NN}}\left(t_{e}^{i}\right)}{N}S^{\text{NN}}\left(t_{e}^{i}\right){I_{c}}^{\text{NN}}\left(t_{e}^{i}\right)+\delta_{q}{I_{q}}^{\text{NN}}\left(t_{e}^{i}\right),\\ L_{5}\left(t_{e}^{i}\right) & =\frac{\text{d}}{\text{dt}}R^{\text{NN}}\left(t_{e}^{i}\right)-\gamma {I_{c}}^{\text{NN}}\left(t_{e}^{i}\right)-\delta_{q}{I_{q}}^{\text{NN}}\left(t_{e}^{i}\right),\\ L_{6}\left(t_{e}^{i}\right) & =\frac{\text{d}}{\text{dt}}{I_{c}}_{\text{cum}}^{\text{NN}}\left(t_{e}^{i}\right)-\frac{\beta c^{\text{NN}}\left(t_{e}^{i}\right)\left(1-q^{\text{NN}}\left(t_{e}^{i}\right)\right)}{N}S^{\text{NN}}\left(t_{e}^{i}\right){I_{c}}^{\text{NN}}\left(t_{e}^{i}\right),\\ L_{7}\left(t_{e}^{i}\right) & =\frac{\text{d}}{\text{dt}}{I_{q}}_{\text{cum}}^{\text{NN}}\left(t_{e}^{i}\right)-\frac{\beta c^{\text{NN}}\left(t_{e}^{i}\right)q^{\text{NN}}\left(t_{e}^{i}\right)}{N}S^{\text{NN}}\left(t_{e}^{i}\right){I_{c}}^{\text{NN}}\left(t_{e}^{i}\right),\\ L_{8}\left(t_{e}^{i}\right) & =\frac{\text{d}}{\text{dt}}{I_{r}}_{\text{cum}}^{\text{NN}}\left(t_{e}^{i}\right)-\frac{\beta c^{\text{NN}}\left(t_{e}^{i}\right)}{N}S^{\text{NN}}\left(t_{e}^{i}\right){I_{c}}^{\text{NN}}\left(t_{e}^{i}\right), \end{array} \end{array}$$

therefore, we have

$$\begin{array}{c} {\text{MSE}}_{ode}=\frac{1}{T_{e}}\sum_{j=1}^{M}\sum_{i=1}^{T_{e}}{\left|L_{j}(t_{e}^{i})\right|}^{2}. \end{array}$$

Finally, we simultaneously learn the network parameters and infer the unknown parameters of the model (1) by training the neural network to minimize the loss function (3). We use TDINN algorithm for fitting and parameter inferring based on the data available in different regions. The algorithm is implemented in Python using Tensorflow [46], an open source library for deep learning computations. We found empirically that the neural network structures used to solve model (1) and inferred time-dependent parameters *c*(*t*) and *q*(*t*) may be different due to the different sample sizes of observed data in various regions. The corresponding depth and width of neural networks are given in Table 2. We use the hyperbolic tangent function *tanh*(*x*) as the activation function *σ* shown in Fig 2. For the optimization of the loss function (3), we use a gradient-based optimizer such as the Adam optimizer [47], whose learning rate is set to be 0.001 by default, and the number of training iterations for each region is listed in Table 2.

**Table 2:** Hyperparameters for the problems in this study.

Regions	Xi’an	Guangzhou	Yangzhou	Hainan	Xinjiang	Liaoning
Hyperparameters						
NN depth and width for *U*(*t*)	(5, 64)	(5, 50)	(10, 64)	(3, 32)	(7, 32)	(7, 32)
NN depth and width for *c*(*t*)	(1, 10)	(1, 10)	(1, 20)	(1, 16)	(1, 16)	(3, 16)
NN depth and width for *q*(*t*)	(1, 10)	(1, 10)	(1, 20)	(1, 16)	(1, 16)	(3, 16)
Learning rate	0.001	0.001	0.001	0.001	0.001	0.001
Iterations	2 × 10^4^	3 × 10^4^	3 × 10^4^	1 × 10^4^	1 × 10^4^	3 × 10^4^

## Results

### Model calibration

**Fig 3:**
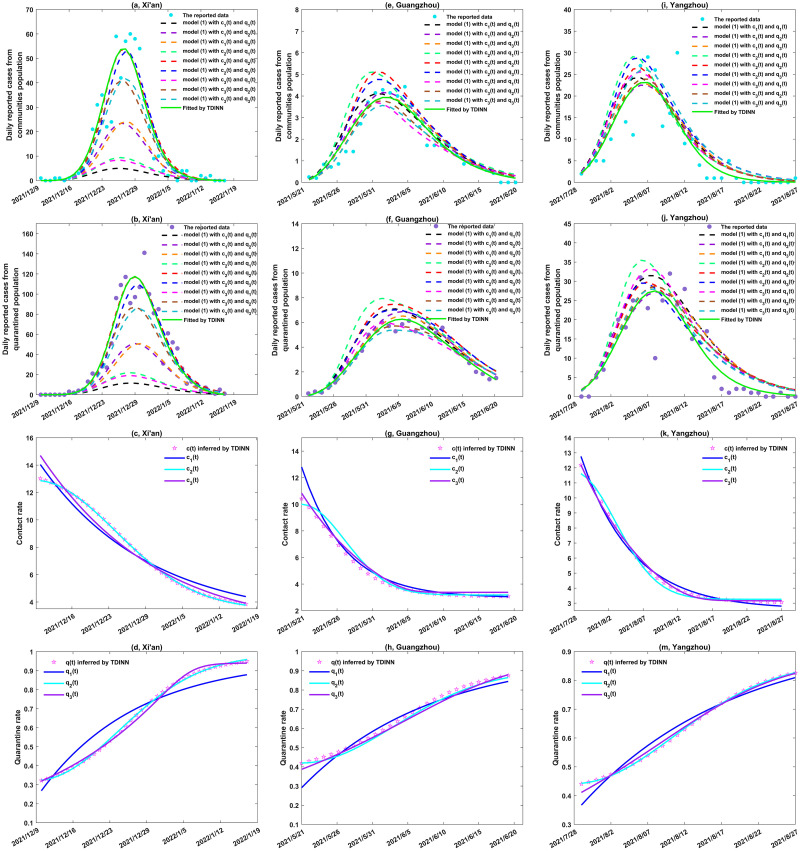
Data fitting and inference of the time-dependent parameters by TDINN algorithm for the local outbreaks in Xi’an, Guangzhou, and Yangzhou. (a)-(b), (e)-(f) and (i)-(j) show the fitting results in Xi’an, Guangzhou and Yangzhou, respectively, where the cyan and purple solid dots represent the daily reported data from communities and quarantined population respectively, green solid curves represent the best fitting results by TDINN, the dashed curves represent the corresponding solution curves after substituting various combinations of the family of functions (4) and (5) into model (1). (c)-(d), (g)-(h) and (k)-(m) show the inference and fitting results of the time-dependent contact rate *c*(*t*) and quarantined rate *q*(*t*) in Xi’an, Guangzhou and Yangzhou, respectively, where the magenta pentagrams represent the inference results of *c*(*t*) and *q*(*t*) by TDINN and the solid curves represent the fitting results of *c*(*t*) and *q*(*t*) based on different functions in (4) and (5).

**Fig 4:**
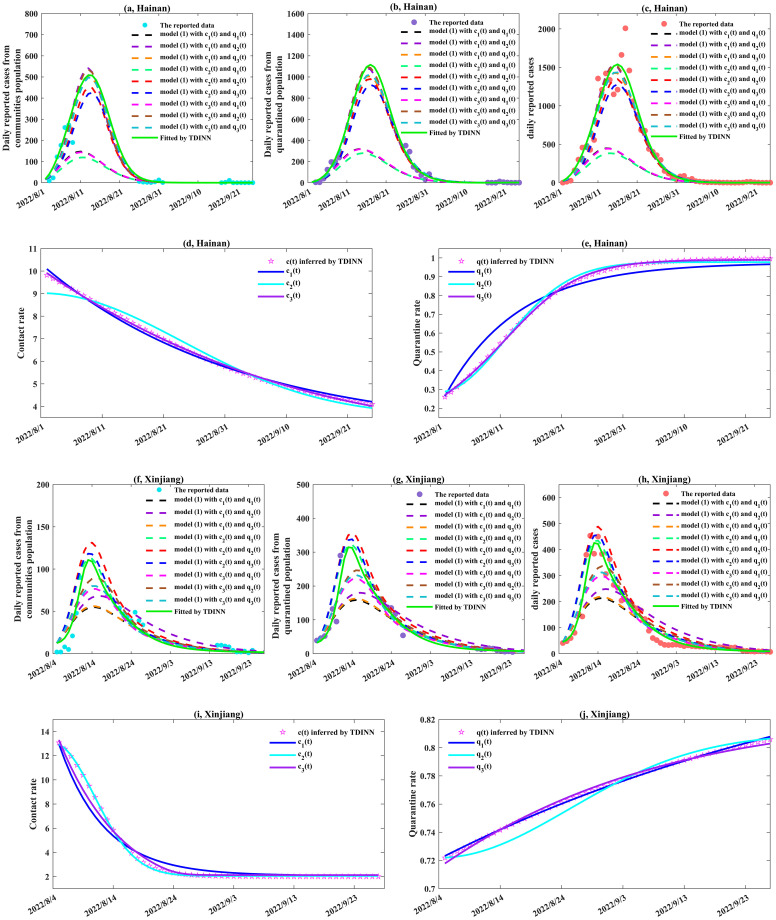
Data fitting and inference of the time-dependent parameters by TDINN algorithm for the local outbreaks in Hainan and Xinjiang. (a)-(c) and (f)-(h) show the fitting results in Hainan and Xinjiang, respectively, where the cyan solid dots represent the daily reported data from communities, the purple solid dots represent the daily reported data from quarantined population and the red solid dots represent the daily reported data, green solid curves represent the best fitting results by TDINN, the dashed curves represent the corresponding solution curves after substituting various combinations of the family of functions (4) and (5) into model (1). (d)-(e) and (i)-(j) show the inference and fitting results of the time-dependent contact rate *c*(*t*) and quarantined rate *q*(*t*) in Hainan and Xinjiang, respectively, where the magenta pentagrams represent the inference results of *c*(*t*) and *q*(*t*) by TDINN and the solid curves represent the fitting results of *c*(*t*) and *q*(*t*) based on different functions in (4) and (5).

For Xi’an, Guangzhou and Yangzhou, we fitted the daily reported cases from communities and from quarantined zone through the TDINN algorithm, while for Hainan and Xinjiang, we further fitted daily reported cases. We present the best fitting results in Figs 3a, 3b, 3e, 3f, 3i, 3j, 4a–4c and 4f–4h (green solid lines), and the inferred time-dependent parameters *c*(*t*) and *q*(*t*) for each region in Figs 3c, 3d, 3g, 3h, 3k, 3m, 4d, 4e, 4i and 4j (magenta pentagrams), respectively. In addition, the estimated parameter values are listed in Table 1.

As we can see from Figs 3 and 4, the TDINN algorithm can fit the daily reported cases from communities and from quarantined zones very well, and can also automatically capture the temporal variations of contact rate and quarantine rate under different epidemic patterns for different regions. It is worth noting that although part of data on the daily confirmed cases from communities and from quarantined zone in Hainan and Xinjiang are available, our algorithm can still accurately simulate the complete epidemic evolution trend for two regions. These numerical simulation results indicate that the proposed TDINN algorithm can not only adapt well to multi-source epidemic data in different regions, but also extract relevant information that can quantify the intensity of control interventions. Moreover, the TDINN algorithm can infer the unobserved dynamics of epidemic based on sparse and noisy observation data, thereby reconstructing the complete epidemic development process.

It is worth noting here that although we do not have any prior information on the contact rate *c*(*t*) and quarantine rate *q*(*t*), that is, we do not assume the specific function expressions for *c*(*t*) and *q*(*t*) in advance, the variations in the contact rate and quarantine rate over time in different regions can completely be extracted from the multi-source epidemic data. From Figs 3c, 3d, 3g, 3h, 3k, 3m, 4d, 4e, 4i and 4j (magenta pentagrams), we can find that *c*(*t*) and *q*(*t*) inferred by TDINN algorithm show regional dependent, that is, the temporal evolution curves of *c*(*t*) and *q*(*t*) corresponding to different regions show significantly different behaviors in terms of shape, indicating differences in strength of implementation and execution of control intervention strategies to alleviate the COVID-19 infection in each region. This difference makes the epidemic curves in various regions exhibit diversity in terms of peak values and peak times, which further demonstrating the importance of capturing the underlying efficacy of intervention to quickly realize dynamic zero-case policy at that time.

In addition, based on the inferred *c*(*t*) and *q*(*t*), we find that contact rate *c*(*t*) shows a downward trend (shown in Figs 3c, 3g, 3k, 4d and 4i, while quarantine rate *q*(*t*) shows an upward trend (shown in Figs 3d, 3h, 3m, 4e and 4j. This is associated with the fact that once an outbreak taking off, China’s dynamic zero-case policy leads to an increase in the quarantine rate and the contact rate decline due to local lockdown and the enhanced close contact tracing and quarantine measure. Then, an interesting question raised from this observation is whether we can describe the temporal evolution patterns of *c*(*t*) and *q*(*t*) with specific functions to better quantify the evolution of the interventions, and consequently enhance the interpretability of deep learning.

### Interpretability analysis of time-dependent parameters

Note that the contact rate and quarantine rate resulting from TDINN inference are two abstract time series without particular expressions. Therefore, it is worth formulating the appropriate functions for contact rate and quarantine rate, which can describe deep learning’s inference results and reveal the temporal evolution process of interventions. These functions not only aid in better understanding the behavior of deep learning during the inference process, but also improve the prediction accuracy and interpretability of model, providing guidance for designing more effective prevention and control strategies.

Note that the increasing/decreasing pattern of time series may be associated with various formulas of rate functions. Here, we consider the contact rate *c*(*t*) and quarantine rate *q*(*t*) as a family of functions, with each family comprising three distinct forms denoted as *c*_1_(*t*), *c*_2_(*t*), *c*_3_(*t*) and *q*_1_(*t*), *q*_2_(*t*), *q*_3_(*t*), respectively. The explicit expressions for these functions are assumed as follows:

$$\begin{array}{c} \left\{ \begin{array}{cc} c_{1}\left(t\right) & =(c_{01}-c_{b1})e^{-r_{11}t}+c_{b1},\\ c_{2}\left(t\right) & =(c_{02}-c_{b2})e^{-{\left(r_{12}t\right)}^{2}}+c_{b2},\\ c_{3}\left(t\right) & =c_{b3}[1+({\left(\frac{c_{b3}}{c_{03}}\right)}^{-m}-1)e^{-r_{13}mt}{]}^{\frac{1}{m}}, \end{array}\right. \end{array}$$ (4)

and

$$\begin{array}{c} \left\{ \begin{array}{cc} q_{1}\left(t\right) & =(q_{01}-q_{m1})e^{-r_{21}t}+q_{m1},\\ q_{2}\left(t\right) & =(q_{02}-q_{m2})e^{-{\left(r_{22}t\right)}^{2}}+q_{m2},\\ q_{3}\left(t\right) & =q_{m3}[1+({\left(\frac{q_{03}}{q_{m3}}\right)}^{-n}-1)e^{-r_{23}nt}{]}^{-\frac{1}{n}}. \end{array}\right. \end{array}$$ (5)

Here, the functions *c*_1_(*t*) and *q*_1_(*t*) are derived from existing literatures [48–50]. Parameter *c*_0*i*_ is the initial contact rate, parameter *c*_*bi*_ represents the minimum contact rate, and parameter *r*_1*i*_ denotes the exponential decreasing rate of the contact rate, *i* = 1, 2, 3. Parameter *q*_0*i*_ is the initial quarantine rate, parameter *q*_*mi*_ denotes the maximum quarantine rate with the intervention being implemented, and parameter *r*_2*i*_ represents the exponential increasing rate of quarantine rate, *i* = 1, 2, 3. In contrast to the exponential decay/increasing functions of *c*_1_(*t*) and *q*_1_(*t*), the sustained strengthening control strategies is described by the Gaussian decay functions [51] of *c*_2_(*t*) and *q*_2_(*t*). Additionally, the construction of *c*_3_(*t*) and *q*_3_(*t*) is based on the analytical solution of the Rosenzweig model [52], where *m* and *n* are interference constants. Then, an interesting question is which function in the family of functions (4) and (5) can accurately describe the temporal evolution trends of *c*(*t*) and *q*(*t*) inferred by TDINN.

To address this question, we initially begin by considering the time series corresponding to *c*(*t*) and *q*(*t*) learned from the TDINN algorithm as observed data, denoting them as $\hat{c}\left(t\right)$ and $\hat{q}\left(t\right)$, respectively, where $t=1,2,\cdots ,T_{d}$. Next, we fit the functions in (4) and (5) to the observed data $\hat{c}\left(t\right)$ and $\hat{q}\left(t\right)$, estimate the corresponding unknown parameters, and select the appropriate function formula based on the statistical criterion. This is equivalent to solving the optimization problem:

$$\begin{array}{c} \underset{\theta }{\text{arg}\;\text{min}}\;{L_{c}}_{i}(\theta ),\;\underset{\vartheta }{\text{arg}\;\text{min}}\;{L_{q}}_{i}(\vartheta ),i=1,2,3, \end{array}$$ (6)

with

$${L_{c}}_{i}(\theta )=\sum_{t=1}^{T_{d}}{\left|c_{i}(t,\theta )-\hat{c}(t)\right|}^{2},{L_{q}}_{i}(\vartheta )=\sum_{t=1}^{T_{d}}{\left|q_{i}(t,\vartheta )-\hat{q}(t)\right|}^{2}.$$

Parameters *θ* and *ϑ* represent the unknown parameter vectors in (4) and (5), respectively. Then, we utilize the least squares(LS) method to solve the optimization problem (6), and consequently obtain the estimated values of the unknown parameters in the family of functions (4) and (5), as listed in Table 3. The fitting results for each region are shown in Figs 3c, 3d, 3g, 3h, 3k, 3m, 4d, 4e, 4i and 4j (solid lines), respectively. Finally, we determine the optimal function form to accurately capture the temporal evolution of contact rate *c*(*t*) and quarantine rate *q*(*t*) inferred by the TDINN algorithm based on the criterion of minimizing the root mean squared error (RMSE). We computed the root mean square errors (${\text{RMSE}}_{c_{i}}$ and ${\text{RMSE}}_{q_{i}}$) which are generated by different functions in (4) and (5) when fitting the observed data $\hat{c}\left(t\right)$ and $\hat{q}\left(t\right)$, where

$$\begin{array}{c} {\text{RMSE}}_{c_{i}}=\sqrt{\frac{1}{T_{d}}\sum_{t=1}^{T_{d}}{\left[c_{i}(t)-\hat{c}(t)\right]}^{2}},\;\;{\text{RMSE}}_{q_{i}}=\sqrt{\frac{1}{T_{d}}\sum_{t=1}^{T_{d}}{\left[q_{i}(t)-\hat{q}(t)\right]}^{2}},\;i=1,2,3. \end{array}$$

**Table 3:** Parameter definitions and estimation for functions *c*_*i*_(*t*) and *q*_*i*_(*t*) (i = 1,2,3).

Parameter		Definitions	Estimated values					Sources
			Xi’an	Guangzhou	Yangzhou	Hainan	Xinjiang	
*c*_0*i*_	*c*_01_	Contact rate at the initial time	14.6054	12.7988	14.2205	10.3253	14.5078	Estimated
	*c*_02_		12.8872	10.0039	11.7293	9.0234	13.0733	Estimated
	*c*_03_		15.2903	10.8316	13.2506	10.0992	14.5899	Estimated
*c*_*bi*_	*c*_*b*1_	Minimum contact rate under the current control strategies	2.6624	2.9691	2.5979	2.8232	2.0714	Estimated
	*c*_*b*2_		3.4625	3.1812	3.2593	3.5705	2.0515	Estimated
	*c*_*b*3_		2.5073	3.3792	3.1596	3.0476	2.1241	Estimated
*r*_1*i*_	*r*_11_	Exponential decreasing rate of contact rate	0.0483	0.1703	0.1342	0.0313	0.1328	Estimated
	*r*_12_		0.0463	0.1213	0.1176	0.0306	0.1038	Estimated
	*r*_13_		0.0404	0.0802	0.0836	0.0189	0.0929	Estimated
*m*		Interference constant	2	12	8	4	8	Assumed
*q*_0*i*_	*q*_01_	Quarantined rate at the initial time	0.2299	0.2912	0.3383	0.2020	0.7210	Estimated
	*q*_02_		0.3230	0.4199	0.4416	0.2854	0.7219	Estimated
	*q*_03_		0.3070	0.3870	0.3972	0.2483	0.7149	Estimated
*q*_*mi*_	*q*_*m*1_	Maximum quarantined rate under the current control strategies	0.9633	0.9847	0.9555	0.9744	0.8969	Estimated
	*q*_*m*2_		0.9844	0.9039	0.8642	0.9775	0.8100	Estimated
	*q*_*m*3_		0.9405	0.9695	0.8789	0.9899	0.8233	Estimated
*r*_2*i*_	*r*_21_	Exponential increasing rate of quarantined rate	0.0541	0.0571	0.0481	0.0840	0.0126	Estimated
	*r*_22_		0.0452	0.0566	0.0519	0.0665	0.0332	Estimated
	*r*_23_		0.0388	0.0392	0.0364	0.0911	0.0171	Estimated
*n*		Interference constant	12	4	4	2	2	Assumed

**Fig 5:**
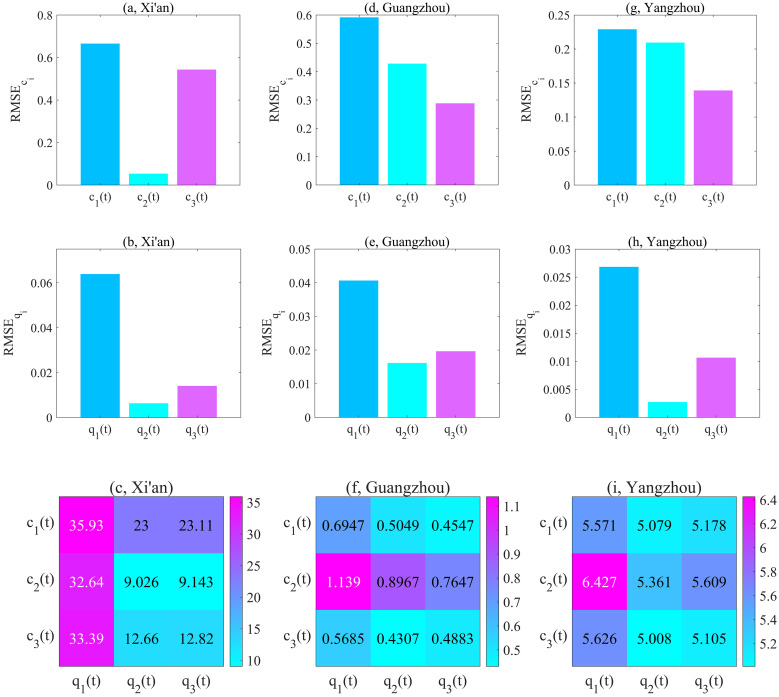
The optimal contact/quarantine rates from the family of functions (4) and (5) for Xi’an, Guangzhou and Yangzhou. (a, d, g) Root mean square error(${\text{RMSE}}_{c_{i}}$), corresponding to fitting the time-dependent contact rate learned by TDINN algorithm using *c*_1_(*t*), *c*_2_(*t*) and *c*_3_(*t*) in Xi’an, Guangzhou and Yangzhou. (b, e, h) Root mean square error(${\text{RMSE}}_{q_{i}}$), corresponding to fitting the time-dependent quarantine rate learned by TDINN algorithm using *q*_1_(*t*), *q*_2_(*t*) and *q*_3_(*t*) in Xi’an, Guangzhou and Yangzhou. (c, f, i) Average root mean square error (${\text{ARMSE}}_{c_{i}}^{q_{j}}i,j=1,2,3$), corresponding to fitting epidemic data using model (1) based on various combinations of the family of functions (4) and (5) in Xi’an, Guangzhou and Yangzhou.

**Fig 6:**
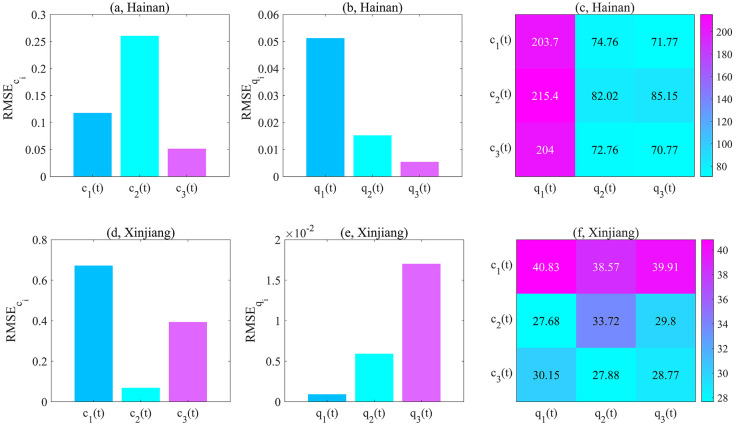
The optimal contact/quarantine rates from the family of functions (4) and (5) for Hainan and Xinjiang. (a, d) Root mean square error(${\text{RMSE}}_{c_{i}}$), corresponding to fitting the time-dependent contact rate learned by TDINN algorithm using *c*_1_(*t*), *c*_2_(*t*) and *c*_3_(*t*) in Hainan and Xinjiang. (b, e) Root mean square error(${\text{RMSE}}_{q_{i}}$), corresponding to fitting the time-dependent quarantine rate learned by TDINN algorithm using *q*_1_(*t*), *q*_2_(*t*) and *q*_3_(*t*) in Hainan and Xinjiang. (c, f) Average root mean square error (${\text{ARMSE}}_{c_{i}}^{q_{j}},i,j=1,2,3$), corresponding to fitting epidemic data using model (1) based on various combinations of the family of functions (4) and (5) in Hainan and Xinjiang.

Note that, the functions with the smallest RMSE*c*_*i*_ and RMSE*q*_*i*_ were selected as the best candidates. According to Figs 5a, 5b, 5d, 5e, 5g, 5h, 6a, 6b, 6d and 6e, we can draw the following conclusions:

- for Xi’an: ${\text{RMSE}}_{c_{1}}>{\text{RMSE}}_{c_{3}}>{\text{RMSE}}_{c_{2}}$, ${\text{RMSE}}_{q_{1}}>{\text{RMSE}}_{q_{3}}>{\text{RMSE}}_{q_{2}}$;
- for Guangzhou: ${\text{RMSE}}_{c_{1}}>{\text{RMSE}}_{c_{2}}>{\text{RMSE}}_{c_{3}}$, ${\text{RMSE}}_{q_{1}}>{\text{RMSE}}_{q_{3}}>{\text{RMSE}}_{q_{2}}$;
- for Yangzhou: ${\text{RMSE}}_{c_{1}}>{\text{RMSE}}_{c_{2}}>{\text{RMSE}}_{c_{3}}$, ${\text{RMSE}}_{q_{1}}>{\text{RMSE}}_{q_{3}}>{\text{RMSE}}_{q_{2}}$;
- for Hainan: ${\text{RMSE}}_{c_{2}}>{\text{RMSE}}_{c_{1}}>{\text{RMSE}}_{c_{3}}$, ${\text{RMSE}}_{q_{1}}>{\text{RMSE}}_{q_{2}}>{\text{RMSE}}_{q_{3}}$;
- for Xinjiang: ${\text{RMSE}}_{c_{1}}>{\text{RMSE}}_{c_{3}}>{\text{RMSE}}_{c_{2}}$, ${\text{RMSE}}_{q_{3}}>{\text{RMSE}}_{q_{2}}>{\text{RMSE}}_{q_{1}}$.

Based on the above results, we can select the optimal functions to quantify the evolution of the interventions in each region, that is, for Xi’an, Guangzhou, Yangzhou, Hainan and Xinjiang, the optimal rate functions are *c*_2_(*t*) and *q*_2_(*t*), *c*_3_(*t*) and *q*_2_(*t*), *c*_3_(*t*) and *q*_2_(*t*), *c*_3_(*t*) and *q*_3_(*t*), *c*_2_(*t*) and *q*_1_(*t*) respectively.

To further validate our conclusions, we substituted various functions into the model (1) and re-fitted the multi-source data for each region by using the estimated parameters in Tables 1 and 3. The fitting results are presented in Figs 3a, 3b, 3e, 3f, 3i, 3j, 4a–4c and 4f–4h (dotted lines). Note that here we consider the average root mean square error (${\text{ARMSE}}_{c_{i}}^{q_{j}}$, *i*, *j* = 1, 2, 3) as a metric to evaluate the fitting performance of model (1) with different function combinations in the family of functions (4) and (5) for multi-source data in each region.

For Xi’an, Guangzhou and Yangzhou,

$$\begin{array}{c} {\text{ARMSE}}_{c_{i}}^{q_{j}}=\frac{1}{2}(\sqrt{\frac{1}{T_{d}}\sum_{t=1}^{T_{d}}{\left[{\tilde{I}}_{c_{\text{new}}}(t)-{I_{c}}_{\text{new}}^{data}(t)\right]}^{2}}+\sqrt{\frac{1}{T_{d}}\sum_{t=1}^{T_{d}}{\left[{\tilde{I}}_{q_{\text{new}}}(t)-I_{q_{\text{new}}}^{data}(t)\right]}^{2}}), \end{array}$$

for Hainan and Xinjiang,

$$\begin{array}{c} \begin{array}{cc} {\text{ARMSE}}_{c_{i}}^{q_{j}}= & \frac{1}{3}(\sqrt{\frac{1}{T_{d}}\sum_{t=1}^{T_{d}}{\left[{\tilde{I}}_{c_{\text{new}}}(t)-{I_{c}}_{\text{new}}^{data}(t)\right]}^{2}}+\sqrt{\frac{1}{T_{d}}\sum_{t=1}^{T_{d}}{\left[{\tilde{I}}_{q_{\text{new}}}(t)-I_{q_{\text{new}}}^{data}(t)\right]}^{2}}\\ & +\sqrt{\frac{1}{T_{d}}\sum_{t=1}^{T_{d}}{\left[{\tilde{I}}_{r_{\text{new}}}(t)-I_{r_{\text{new}}}^{data}(t)\right]}^{2}}), \end{array} \end{array}$$

where ${\tilde{I}}_{c_{\text{new}}}$, ${\tilde{I}}_{q_{\text{new}}}\;{\tilde{I}}_{r_{\text{new}}}$ are the predicted values by solving model (1).

Considering all possible combinations of rate functions, the smaller ${\text{ARMSE}}_{c_{i}}^{q_{j}}$ value indicates the better fitting effect of model (1) on multi-source data. In the following, we summarized how the ${\text{ARMSE}}_{c_{i}}^{q_{j}}$ varied with respect to different choices of contact rate and quarantine rate for each region in Figs 5c, 5f, 5i, 6c and 6f. According to Figs 5c, 5f, 5i, 6c and 6f, the corresponding ${\text{ARMSE}}_{c_{i}}^{q_{j}}$ values for each region exhibit the following relationship:

- for Xi’an: ${\text{ARMSE}}_{c_{1}}^{q_{1}}>{\text{ARMSE}}_{c_{3}}^{q_{1}}>{\text{ARMSE}}_{c_{2}}^{q_{1}}>{\text{ARMSE}}_{c_{1}}^{q_{3}}>{\text{ARMSE}}_{c_{1}}^{q_{2}}>{\text{ARMSE}}_{c_{3}}^{q_{3}}>{\text{ARMSE}}_{c_{3}}^{q_{2}}>{\text{ARMSE}}_{c_{2}}^{q_{3}}>{\text{ARMSE}}_{c_{2}}^{q_{2}}$;
- for Guangzhou: ${\text{ARMSE}}_{c_{2}}^{q_{1}}>{\text{ARMSE}}_{c_{2}}^{q_{2}}>{\text{ARMSE}}_{c_{2}}^{q_{3}}>{\text{ARMSE}}_{c_{1}}^{q_{1}}>{\text{ARMSE}}_{c_{3}}^{q_{1}}>{\text{ARMSE}}_{c_{1}}^{q_{2}}>{\text{ARMSE}}_{c_{3}}^{q_{3}}>{\text{ARMSE}}_{c_{1}}^{q_{3}}>{\text{ARMSE}}_{c_{3}}^{q_{2}}$;
- for Yangzhou: ${\text{ARMSE}}_{c_{2}}^{q_{1}}>{\text{ARMSE}}_{c_{3}}^{q_{1}}>{\text{ARMSE}}_{c_{2}}^{q_{3}}>{\text{ARMSE}}_{c_{1}}^{q_{1}}>{\text{ARMSE}}_{c_{2}}^{q_{2}}>{\text{ARMSE}}_{c_{1}}^{q_{3}}>{\text{ARMSE}}_{c_{3}}^{q_{3}}>{\text{ARMSE}}_{c_{1}}^{q_{2}}>{\text{ARMSE}}_{c_{3}}^{q_{2}}$;
- for Hainan: ${\text{ARMSE}}_{c_{2}}^{q_{1}}>{\text{ARMSE}}_{c_{3}}^{q_{1}}>{\text{ARMSE}}_{c_{1}}^{q_{1}}>{\text{ARMSE}}_{c_{2}}^{q_{3}}>{\text{ARMSE}}_{c_{2}}^{q_{2}}>{\text{ARMSE}}_{c_{1}}^{q_{2}}>{\text{ARMSE}}_{c_{3}}^{q_{2}}>{\text{ARMSE}}_{c_{1}}^{q_{3}}>{\text{ARMSE}}_{c_{3}}^{q_{3}}$;
- for Xinjiang: ${\text{ARMSE}}_{c_{1}}^{q_{1}}>{\text{ARMSE}}_{c_{1}}^{q_{3}}>{\text{ARMSE}}_{c_{1}}^{q_{2}}>{\text{ARMSE}}_{c_{2}}^{q_{2}}>{\text{ARMSE}}_{c_{3}}^{q_{1}}>{\text{ARMSE}}_{c_{2}}^{q_{3}}>{\text{ARMSE}}_{c_{3}}^{q_{3}}>{\text{ARMSE}}_{c_{3}}^{q_{2}}>{\text{ARMSE}}_{c_{2}}^{q_{1}}$.

The above results show that selecting *c*_2_(*t*) and *q*_2_(*t*) (or *c*_3_(*t*) and *q*_2_(*t*), *c*_3_(*t*) and *q*_2_(*t*), *c*_3_(*t*) and *q*_3_(*t*), *c*_2_(*t*) and *q*_1_(*t*)) as the contact rate and quarantine rate leads to the smallest ARMSE value for Xi’an (or Guangzhou, Yangzhou, Hainan, Xinjiang). This indicates that model (1) can accurately replicate the development process of the COVID-19 epidemic in Xi’an(or Guangzhou, Yangzhou, Hainan, Xinjiang) under this combination, which further validates our previous conclusion that *c*_2_(*t*) and *q*_2_(*t*) (or *c*_3_(*t*) and *q*_2_(*t*), *c*_3_(*t*) and *q*_2_(*t*), *c*_3_(*t*) and *q*_3_(*t*), *c*_2_(*t*) and *q*_1_(*t*)) are the optimal functions for quantifying the evolution of control interventions in Xi’an(or Guangzhou, Yangzhou, Hainan, Xinjiang). Based on the optimal rate functions for each region (see Figs 5 and 6 for detail), we can find that it is difficult to construct a universal function combination to quantify the control intervention strategies implemented in different regions. That is to say, in order to response the outbreak, the pattern of epidemic prevention and control in one region cannot be directly applied to another region. Ideally, we should flexibly adjust and develop appropriate prevention and control measures according to the actual situation of different regions.

According to the above analysis, we utilized the time series inferred by the TDINN algorithm to get the optimal contact rate and quarantine rate from the family of functions (4) and (5), which enabled us to accurately quantify the strength of control measures in each region. Further, it is worth noting all parameters in the rate functions have realistic meanings, then the selected rate functions help to enhance the interpretability of the time series inferred by deep learning. In addition, we can achieve the best fitting effect after substituting the optimal contact rate and quarantine rate into the model (1), which further validates that this method of quantifying the dynamic evolution of interventions is feasible. This method can also aid in improving our understanding of how control strategies are dynamically adjusted when fighting against epidemics.

### Simulation of the multiple epidemic waves

**Fig 7:**
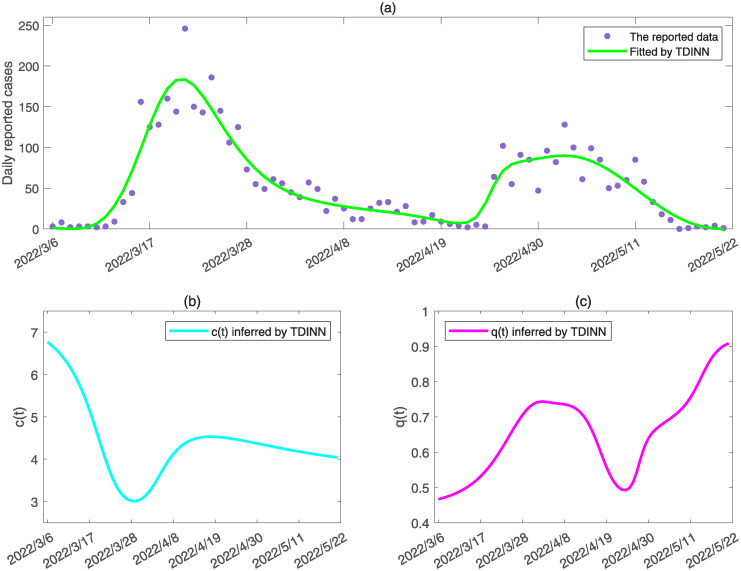
Data fitting and inference of the time-dependent parameters by TDINN algorithm for multiple waves of COVID-19 infection in Liaoning province. (a) shows the fitting results for the available data in Liaoning, where the purple solid dots represent the daily reported data, green solid curves represent the best fitting results by TDINN. (b) and (c) show the inferred time-dependent contact rate *c*(*t*) and quarantine rate *q*(*t*) by TDINN, respectively.

To further illustrate the effectiveness of our proposed method, we also apply the proposed TDINN algorithm to the simulation of multiple waves of COVID-19 infection. To do this, we simulated the dynamics of the epidemic based on daily reported cases in Liaoning province and visualize the simulation results in Fig 7. The simulation results show that the TDINN algorithm can not only fit the epidemic data containing multiple waves well (see Fig 7a), but also capture the information on strengthening and relaxation of intervention measures, that is, the inferred contact rate and quarantine rate exhibit fluctuations as shown in Fig 7b and 7c.

In fact, as the epidemic initially took off, we observed an increase in quarantine rate and a decrease in contact rate due to enhanced intervention measures to mitigate epidemic. While the outbreak was subsiding, the gradual relaxation of control interventions led to the quarantine rate decline and the contact rate increase, and thereby possibly inducing a resurgence of epidemic. As a consequence, comparing the inferred contact rate and quarantine rate with the time series of daily reported cases containing multiple epidemic waves (Fig 7a–7c), we can observe a feedback loop: epidemic taking off → quarantine rate increasing and contact rate decreasing → epidemic subsiding → quarantine rate decreasing and contact rate increasing → epidemic resurging, which drives multiple COVID-19 epidemic waves as observed in Liaoning province. It is worth noting that the inferred time series on contact rate and quarantine rate in Fig 7b and 7c exhibit complicated behaviors, which are difficult to simulate accurately through the family of functions (4) and (5).

## Discussion and conclusion

During the COVID-19 pandemic, control measures played an important role in mitigating the disease spread. In particular, massive contact tracing following prompt quarantine and isolation showed decisive effect in dynamic clearing of the COVID-19 epidemic in China. Hence quantifying the dynamic contact rate and quarantine rate and estimate their impacts remain challenging. In this study, we integrated data-driven deep learning and dynamics-driven first principle modeling, and proposed an extended transmission-dynamics-informed neural network (TDINN) algorithm by encoding SIR-type compartment model into the neural networks, in order to obtain the time-dependent rate functions of mechanistic models. With the developed TDINN algorithm, we simulated the dynamics of COVID-19 infection in Xi’an, Guangzhou, Yangzhou, Hainan, Xinjiang and Liaoning province, by simultaneously inferring the unknown time-independent and time-dependent parameters.

The TDINN algorithm enables us to successfully encode the contact rate and quarantine rate derived from deep neural networks into the compartment model, as well as integrating the transmission dynamic model into the deep neural networks. It is important to note that the TDINN algorithm overcomes some disadvantages of traditional transmission dynamic models for simulating the development process of the COVID-19 epidemic. For example, in the classic compartment model, the contact rate and quarantine rate are usually assumed to be constant or particular time-dependent functions, respectively, to describe the intensities of control interventions [10, 12]. That is, to simulate outbreaks in different regions, we need to pre-set various particular parameter values and/or time-dependent functions to quantify the continuously adjusted control measures in different regions, which significantly limits the performance of the transmission dynamic models. In contrast, our proposed TDINN algorithm can effectively overcome this disadvantage as it associates the transmission dynamic model with deep neural networks through the universal approximation property of neural networks [45] and can capture information on contact rate and quarantine rate from the epidemic data without assuming the particular formula for the rate functions in advance.

Despite the structure of the considered transmission dynamic model (1) in the TDINN algorithm is quite simple, the model (1) incorporates time-dependent contact rate and quarantine rate inferred by neural networks, allowing us to well fit multi-source data for different regions that included daily reported cases in the community and in the quarantined zone (see Fig 3a, 3b, 3e, 3f, 3i and 3j), as well as daily reported cases with multiple epidemic waves (Fig 7). In addition, by using TDINN algorithm we can also reconstruct the epidemic process even if the data are insufficient (Fig 4a–4c and 4f–4h) and obtain the temporal evolution patterns of contact rate *c*(*t*) and quarantine rate *q*(*t*). The estimations of contact/quarantine rates show the regional-dependent (see Figs 3c, 3d, 3g, 3h, 3k, 3m, 4d, 4e, 4i, 4j, 7b and 7c), which indicates that there are differences in efficacy of control intervention strategies adopted in various regions. It further reveals why it is difficult to accurately quantify the strength of control measures through a specific function, that is, pre-setting the particular type of functions may not describe the actual contact rate and quarantine rate.

It is interesting to observe the high consistency in the evolutionary trend of the contact rate and quarantine rate extracted by the TDINN algorithm from a single wave of epidemic (such as Xi’an, Guangzhou, Yangzhou, Hainan and Xinjiang), where the contact rate gradually decreases and the quarantine rate gradually increases (shown in Figs 3c, 3d, 3g, 3h, 3k, 3m, 4d, 4e, 4i and 4j). This suggests that reducing the contact rate and/or increasing the quarantine rate can significantly be associated with decrease in the daily reported cases, which agrees well with previous studies [53, 54]. In addition, Liaoning outbreak experienced two epidemic waves, which is related with the continuous strengthening and relaxation of control interventions, corresponding to the oscillations of the contact and quarantine rates (shown in Fig 7). In return, the shifting of the contact and quarantine rates can also affect the transmission dynamics of COVID-19 pandemic. This generates a feedback loop between the changes in the intensity of control measures and the epidemic shifting, which is the key to drive the fluctuations of the epidemics and is in line with observations of the existing study [11].

A key highlight of this study is that we can select the best combination from a family of functions (4) and (5) to accurately simulate the time series for contact and quarantine rate (*c*(*t*) and *q*(*t*)) learned by TDINN algorithm (Figs 5 and 6). The selection enables us to comprehensively explore the evolution trend of COVID-19 epidemic outbreak in different regions, and study the impact of various intervention strategies on the spread of infectious diseases. In addition, the selected rate functions based on the time series inferred by deep learning have reasonable meanings.

In this study, we proposed the TDINN algorithm, which not only extends the traditional transmission dynamic model by embedding the time-dependent functions learned from the deep neural network, but also extends the neural network by embedding the information of the transmission dynamic model. The novel approach enables us to well integrate the advantages of the transmission mechanism model and the deep neural network. Compared with traditional dynamic models, the TDINN algorithm has better data learning ability and inference ability of unknown rate functions. Compared with end-to-end deep learning, our main results are more interpretable due to the incorporation of known propagation mechanisms. Furthermore, this method can be easily extended to more complex compartment models to study other aspects of emerging infectious diseases.

Our study has some limitations. The transmission dynamic model (1) we considered is fairly simple and may overlook the impact of important factors such as the capacity of healthcare infrastructure, behavioral responses to epidemics, and vaccination on the development of the COVID-19 infection, but we hope the approaches, integrating transmission dynamics with deep learning, are able to be applied more generally. In addition, for the contact rate and quarantine rate inferred from the multiple epidemic waves, it is difficult to accurately simulate their temporal evolution patterns through smooth functions. We leave this for future work.
